# P01.14. Blood lead levels of children using traditional Indian medicine and cosmetics: a feasibility study

**DOI:** 10.1186/1472-6882-12-S1-P14

**Published:** 2012-06-12

**Authors:** J Keosaian, T Venkatesh, P Gardiner, R Saper

**Affiliations:** 1 Boston Medical Center, Boston, USA; 2 National Referral Centre for Lead Poisoning in India, Bengaluru, India

## Purpose

Traditional Indian or Ayurvedic medicines and cosmetics may contain lead. The relationship between cosmetic use (e.g. kohl) and blood lead levels (BLLs) in children has been well documented; however the impact of Ayurvedic use on BLL remains unclear. To begin to address this question, we conducted a pilot study to assess the feasibility of collecting BLLs in children attending Ayurvedic hospitals in India.

## Methods

Our study took place over five days in the summer of 2010 at a large public Ayurveda hospital and a small pediatric clinic in southern India, facilitated by the assistance and buy-in of local community leaders. Using trained interpreters, we administered to parents of pediatric outpatients a standardized questionnaire in Malayalam assessing sociodemographics, Ayurvedic medicine use, kohl use (a traditional cosmetic product) and other potential risk factors for lead exposure. We measured BLL using a LeadCare© II portable lead analyzer.

## Results

The study enrolled 29 children (average age 3.8 years, sd 3.1). Seventy-five percent of children used Ayurvedic medicine in the past two years and 55% reported kohl use. The mean BLL for all children was 6.7 μg/dL (sd 3.5, range 3.5-20.2). The difference in BLLs between Ayurvedic users and non-users was not found to be statistically significant (6.2 μg/dL, sd 2.8 vs. 8.5 μg/dL, sd 5.2, p=0.31). Kohl users had a higher BLL than non-users (7.9 μg/dL vs. 5.3 μg/dL; p=0.03).

## Conclusion

This study demonstrates that is feasible to collect BLLs in pediatric Ayurvedic outpatient clinics in India. Relationships with community members and hospital staff were essential to conducting the study. Results suggest a relationship between kohl use and elevated blood lead levels consistent with other research findings. Larger studies are needed to investigate whether Ayurveda use is an independent risk factor for elevated BLL among Indian children.

